# Hypomyelinating Leukodystrophy 14 (HLD14)-Related UFC1 p.Arg23Gln Decreases Cell Morphogenesis: A Phenotype Reversable with Hesperetin

**DOI:** 10.3390/medicines12010002

**Published:** 2025-01-16

**Authors:** Yuri Ichihara, Maho Okawa, Minori Minegishi, Hiroaki Oizumi, Masahiro Yamamoto, Katsuya Ohbuchi, Yuki Miyamoto, Junji Yamauchi

**Affiliations:** 1 Laboratory of Molecular Neurology, Tokyo University of Pharmacy and Life Sciences, Tokyo 192-0392, Japanmiyamoto-y@ncchd.go.jp (Y.M.); 2 Tsumura Research Laboratories, Tsumura & Co., Ibaraki 200-1192, Japan; ooizumi_hiroaki@mail.tsumura.co.jp (H.O.); hirokoma@h.email.ne.jp (M.Y.); oobuchi_katsuya@mail.tsumura.co.jp (K.O.); 3 Laboratory of Molecular Pharmacology, National Research Institute for Child Health and Development, Tokyo 157-8535, Japan; 4 Diabetic Neuropathy Project, Tokyo Metropolitan Institute of Medical Science, Tokyo 156-8506, Japan

**Keywords:** HLD14, UFC1, hesperetin, oligodendrocyte, differentiation, morphogenesis

## Abstract

Introduction: In the central nervous system (CNS), proper interaction between neuronal and glial cells is crucial for the development of mature nervous tissue. Hypomyelinating leukodystrophies (HLDs) are a group of genetic CNS disorders characterized by hypomyelination and/or demyelination. In these conditions, genetic mutations disrupt the biological functions of oligodendroglial cells, which are responsible for wrapping neuronal axons with myelin sheaths. Among these, an amino acid mutation of the ubiquitin-fold modifier conjugating enzyme 1 (UFC1) is associated with HLD14-related disease, characterized by hypomyelination and delayed myelination in the brain. UFC1 is a critical component of the UFMylation system, functioning similarly to E2-conjugating enzymes in the ubiquitin-dependent protein degradation system. Methodology: We describe how a missense mutation in UFC1 (p.Arg23Gln) leads to the aggregation of UFC1 primarily in lysosomes in FBD-102b cells, which are undergoing oligodendroglial cell differentiation. Results: Cells with mutated UFC1 exhibit reduced Akt kinase phosphorylation and reduced expression of differentiation and myelination marker proteins. Consistently, these cells exhibit impaired morphological differentiation with a reduced ability to extend widespread membranes. Interestingly, hesperetin, a citrus flavonoid with known neuroprotective properties, was found to restore differentiation abilities in cells with the UFC1 mutation. Conclusions: These findings indicate that the HLD14-related mutation in UFC1 causes its lysosomal aggregation, impairing its morphological differentiation. Furthermore, the study highlights potential therapeutic insights into the pathological molecular and cellular mechanisms underlying HLD14 and suggests hesperetin as a promising candidate for treatment.

## 1. Introduction

In the development of the central nervous system (CNS), neuronal cells interact with each other and with glial cells, such as oligodendrocytes (also called oligodendroglial cells) and astrocytes, to form mature brain and spinal cord tissues [1,2]. Specifically, oligodendroglial cells closely associate with neuronal cells by wrapping their axons with myelin sheaths formed from differentiated plasma membranes [1,2,3,4]. These mature myelin sheaths not only enable saltatory conduction but also protect axons from physical and physiological stresses while providing essential nutrients to neuronal cells [1,2]. Abnormalities during the differentiation stage of cells, including oligodendroglial cells, can impede the proper development of mature neuronal tissues [1,2,3,4,5,6], potentially triggering disease [1,2,3,4,5,6].

Pelizaeus–Merzbacher disease is a prototypical disorder among congenital hypomyelinating leukodystrophies (HLDs) and is classified as HLD type 1 (HLD1) [7,8,9,10]. HLDs are syndromes characterized by insufficient white matter formation due to defective myelin sheath development in the CNS. Symptoms of HLDs include nystagmus, developmental delay from birth, spastic quadriplegia, ataxia, and dystonia [7,8,9,10]. Despite the severity of these symptoms, current treatments are limited to symptomatic approaches such as anticonvulsants and muscle relaxants [7,8,9,10,11]. These therapies do not address the root causes of HLDs. A significant challenge in developing targeted treatments is the insufficient understanding of the molecular and cellular mechanisms underlying HLDs [7,8,9,10,11,12]. Additionally, the diverse symptoms across the 28 identified types of HLDs further complicate efforts to identify common disease mechanisms.

Ubiquitin-fold modifier conjugating enzyme 1 (UFC1) functions similarly to E2-conjugating enzymes in the ubiquitin-dependent protein degradation system [13,14,15,16]. UFM1, a ubiquitin-like modifier [13,14,15,16], attaches post-translationally to lysine residues on target proteins in a process similar to ubiquitin conjugation [17,18,19,20]. Despite limited amino acid sequence homology, ubiquitin and UFM1 share similar tertiary structures and conceptual similarities [17,18,19,20]. The enzymes involved in UFMylation, similar to ubiquitin-conjugating enzymes, include UFM1-activating enzyme 5 (UBA5), UFC1, and UFM1-specific ligase 1 (UFL1), which correspond to E1, E2, and E3 enzymes, respectively [13,14,15,16,17,18,19,20]. It is of note that UFM1 and its associated pathway components are evolutionarily conserved [13,14,15,16,17,18,19,20]. Although they are present in almost all eukaryotes, the functional significance of the UFM1 pathway remains to be clarified [21,22].

Mutations in components of the UFM1 pathway are associated with several diseases, including HLD type 14 (HLD14) [23,24,25,26,27,28]. In this study, we first asked whether an HLD14-associated amino acid mutation, p.Arg23Gln (R23Q) in UFC1 [26], causes insufficient and/or abnormal morphogenesis of oligodendroglial cells, possibly leading to hypomyelination. To explore this, we utilized the oligodendroglial cell line FBD-102b, a useful model to observe oligodendroglial cell-like morphological differentiation [29,30]. Additionally, we explored whether the citrus flavonoid hesperetin [31,32,33,34,35,36,37,38] has the potential to mitigate the mutated UFC1-induced phenotypes. These results may shed light on the molecular and cellular pathological mechanisms underlying mutated UFC1 in HLD14-related diseases.

## 2. Materials and Methods

### 2.1. Antibodies, Chemicals and Plasmids

The key antibodies, chemicals, and plasmids used in this study are listed in Table 1.

### 2.2. Cell Culture

The FBD-102b cell line (Riken, Saitama, Japan), a mouse oligodendroglial precursor cell line, was cultured on cell and tissue culture dishes (Nunc brand, ThermoFisher Scientific, Waltham, MA, USA) in Dulbecco’s modified Eagle medium (DMEM)/F-12 medium (DMEM/F-12, Fujifilm, Tokyo, Japan; Nacalai Tesque, Kyoto, Japan) supplemented with 10% heat-inactivated fetal bovine serum (FBS) and PenStrep mixture (ThermoFisher Scientific) in 5% CO_2_ at 37 °C.

To induce morphological differentiation, cells were plated on culture dishes coated with polylysine (Fujifilm; Nacalai Tesque), which provides a highly positive charge. Differentiation was induced by culturing the cells in medium containing 1% FBS for 0 to 5 days in 5% CO_2_ at 37 °C. Cells displaying secondary branches extending from primary ones or myelin-like widespread membranes (sufficiently large to encompass a circle with a diameter greater than 25 micrometers) were classified as differentiated phenotypes [30]. Under these conditions, trypan blue (Nacalai Tesque)-positive attached cells accounted for less than 5% of the population [29,30]. Cell morphologies were visualized using a microscopic system equipped with i-NTER LENS (Micronet, Saitama, Japan). Representative images from multiple samples were analyzed with Image J software ver. Java 8 (https://imagej.nih.gov/ accessed on 23 January 2024).

### 2.3. Transfection

Cells were transfected with the respective plasmids using the ScreenFect A or ScreenFect A Plus transfection kit (Fujifilm) in accordance with the manufacturer’s instructions. The medium was replaced 4 h after transfection. Cells were typically used for biochemical experiments more than 48 h post-transfection [29,30].

Stable clones were collected in the presence of 1 mg/mL of G418 (Fujifilm; Nacalai Tesque) in accordance with the manufacturer’s instructions. Under these conditions, trypan blue positive-attached cells were estimated to constitute less than 5% of the population in each experiment [29,30].

### 2.4. Immunofluorescence

Cells were transfected with the respective plasmids. Coverslip-adherent cells were fixed with 4% paraformaldehyde (Nacalai Tesque) or 100% cold methanol (Nacalai Tesque), blocked with Blocking One (Nacalai Tesque), and incubated with primary antibodies preloaded with fluorescent dye-conjugated secondary antibodies. Samples were then mounted using the Vectashield kit (Vector Laboratories, Burlingame, CA, USA). Fluorescent images were collected using an FV3000 microscope system equipped with a laser-scanning Fluoview apparatus (Olympus, Tokyo, Japan). The images presented in the figures are representative of multiple images merged and analyzed using Image J software.

### 2.5. Cell Lysis, Polyacrylamide Gel Electrophoresis and Immunoblotting

Cells were lysed in lysis buffer containing 50 mM HEPES-NaOH, pH 7.5, 150 mM NaCl, 3 mM MgCl_2_, 1 mM dithiothreitol, 1 mM phenylmethane sulfonylfluoride, 1 microgram/mL leupeptin, 1 mM EDTA, 1 mM Na_3_VO_4_, 10 mM NaF, and 0.5% NP-40 [30].

For denatured conditions, cell lysates were treated with sample buffer (Nacalai Tesque). For non-denaturing conditions, lysates were mixed with non-denaturing sample buffers (Nacalai Tesque). The samples were separated using premade sodium dodecyl sulfate–polyacrylamide gel (Nacalai Tesque; Fujifilm). The electrophoretically separated proteins were transferred to a polyvinylidene fluoride membrane (Fujifilm), blocked with Blocking One, and immunoblotted with primary antibodies followed by peroxidase enzyme-conjugated secondary antibodies. Immunoreactive bands were visualized using the CanoScan LiDE 400 system (Canon, Tokyo, Japan). Multiple experiments were conducted for immunoblotting studies. Band quantification, normalized to another sample’s band set at 100%, was performed using Image J software.

### 2.6. Statistical Analysis

Values are presented as means ± standard deviation (SD) from independent experiments. Intergroup comparisons were performed using the unpaired *t*-test with either Student’s or Welch’s correction in Excel software ver. 2022 (Microsoft, Redmond, WA, USA). Differences were considered statistically significant at *p* < 0.05. All analyses were performed with investigators blinded to sample conditions.

### 2.7. Ethics Statement

Genetically modified cell techniques and related techniques were carried out in accordance with a protocol approved by the Tokyo University of Pharmacy and Life Sciences Gene and Animal Care Committee (Approval Nos. LS28-20 and LSR3-011, 2 April 2024).

## 3. Results

### 3.1. Mutated UFC1 Protein Preferentially Aggregates in the Lysosome

**Figure 1:**
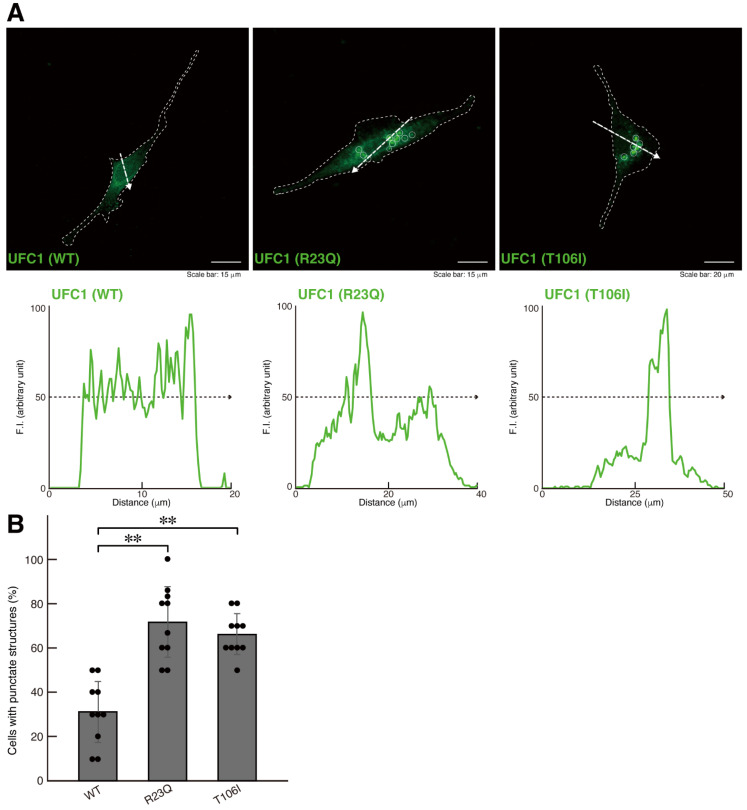
The R23Q and T106I mutant proteins of UFC1 are localized in punctate structures. (**A**,**B**) FBD-102b cells, delineated by dotted lines, were transfected with the plasmid encoding GFP-tagged wild-type (WT) UFC1 or the R23Q or T106I mutant constructs. Transfected cells were detected using transfected proteins (green). White circles in the images showing mutant proteins indicate representative aggregate-like structures (indicated representatively with white circles). Scan plots were performed along the white dotted lines in the direction of the arrows in the images. Graphs showing the fluorescence intensities (arbitrary units) along the white dotted lines in the direction of the arrows are presented in the bottom panels. Percentages of cells with punctate structures were statistically assessed (** *p* < 0.01; *n* = 10 fields [a total of 120 cells per experiment]).

**Figure 2:**
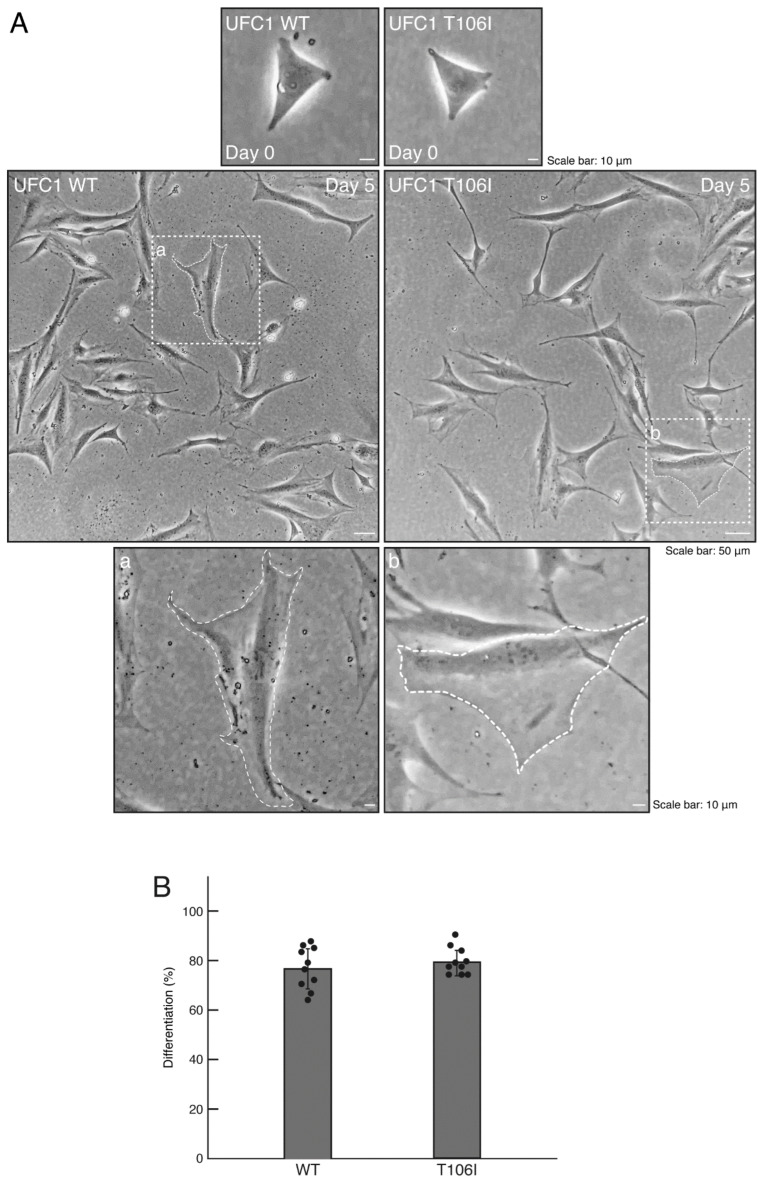
Cells harboring UFC1 with the T106I mutation undergo morphological changes. (**A**,**B**) Cells harboring GFP-tagged wild-type (WT) UFC1 or UFC1 (T106I) were allowed to differentiate for 0 or 5 days. The square fields a and b indicated by the dotted lines in the center panels are magnified in the bottom panels a and b. Cells with widespread membranes were statistically assessed (*n* = 10 fields [a total of 360 cells per experiment]).

To investigate whether UFC1 protein with the R23Q or T106I mutation [26] is localized in the cytoplasm, we transfected mammalian cell expression plasmids encoding these mutated UFC1 proteins into FBD-102b cells. While wild-type UFC1 was localized in the cytoplasmic region, the mutated UFC1 proteins formed aggregate-like structures (Figure 1A). Among the mutations studied, cells expressing UFC1 protein with the R23Q mutation exhibited more aggregate-like structures than those expressing UFC1 protein with the T106I mutation (Figure 1B). Furthermore, cells harboring UFC1 with the T106I mutation displayed a differentiated phenotype that was proportional to the levels of aggregate-like structures (Figure 2A,B). Based on these observations, we focused our subsequent studies on UFC1 protein with the R23Q mutation.

**Figure 3:**
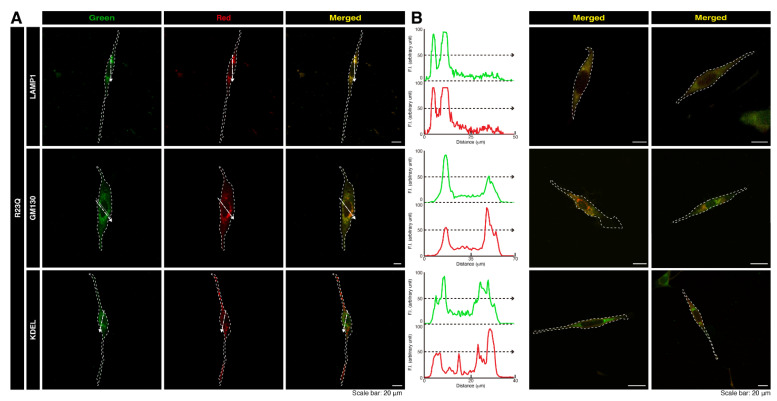
The R23Q mutant protein of UFC1 is primarily co-localized with the lysosome antigen. (**A**,**B**) FBD-102b cells were transfected with the plasmid encoding GFP-tagged UFC1 (R23Q). Cells were detected with transfected proteins (green) and the organelle antigen (red). Scan plots were performed along the white dotted lines in the direction of the arrows in the representative color images (green and red images). Staining data for the other two cells are depicted as merged images in the two lanes on the right.

**Figure 4:**
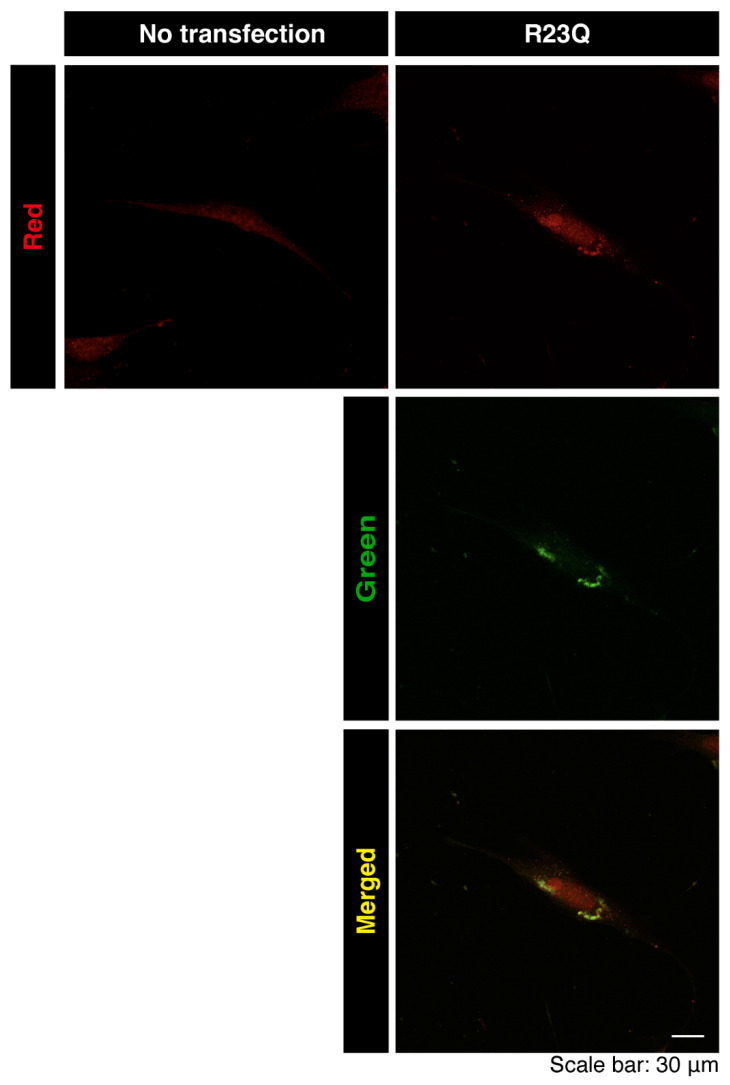
Aggregate-like structures of UFC1 with the R23Q mutation are co-localized with endogenous UFC1. Cells were transfected with or without the plasmid encoding GFP-tagged UFC1 (green) with the R23Q mutation and stained with an anti-UFC1 antibody (red). Merged images are shown as representative examples.

Next, we aimed to identify the organelle containing these aggregate-like structures. We transfected cells with a plasmid encoding UFC1 protein with the R23Q mutation and stained them with major organelle markers. UFC1 protein with the R23Q mutation co-localized with the lysosomal antigen lysosomal-associated membrane protein 1 (LAMP1) (Figure 3A,B). The mutated UFC1 protein was also partially co-localized with the Golgi body antigen GM130 but not with the endoplasmic reticulum (ER) antigen Lys-Asp-Glu-Leu (KDEL). Endogenous UFC1 protein was localized in the cytoplasmic region and appeared to be at least partially incorporated into aggregate-like structures in the presence of the R23Q mutated protein (Figure 4). In contrast, transfected wild-type UFC1 protein did not significantly co-localize with any of the examined organelle antigens (Figure S1).

Since the UFC1 protein with the R23Q mutation appeared to aggregate in the lysosome, we further investigated whether the mutated protein could form aggregated structures. We performed polyacrylamide gel electrophoresis under non-denaturing conditions and confirmed that the mutated protein formed dimeric or possibly oligomeric structures (Figure S2).

### 3.2. UFC1 with the R23Q Mutation Decreases Oligodendroglial Cell Morphological Differentiation

**Figure 5:**
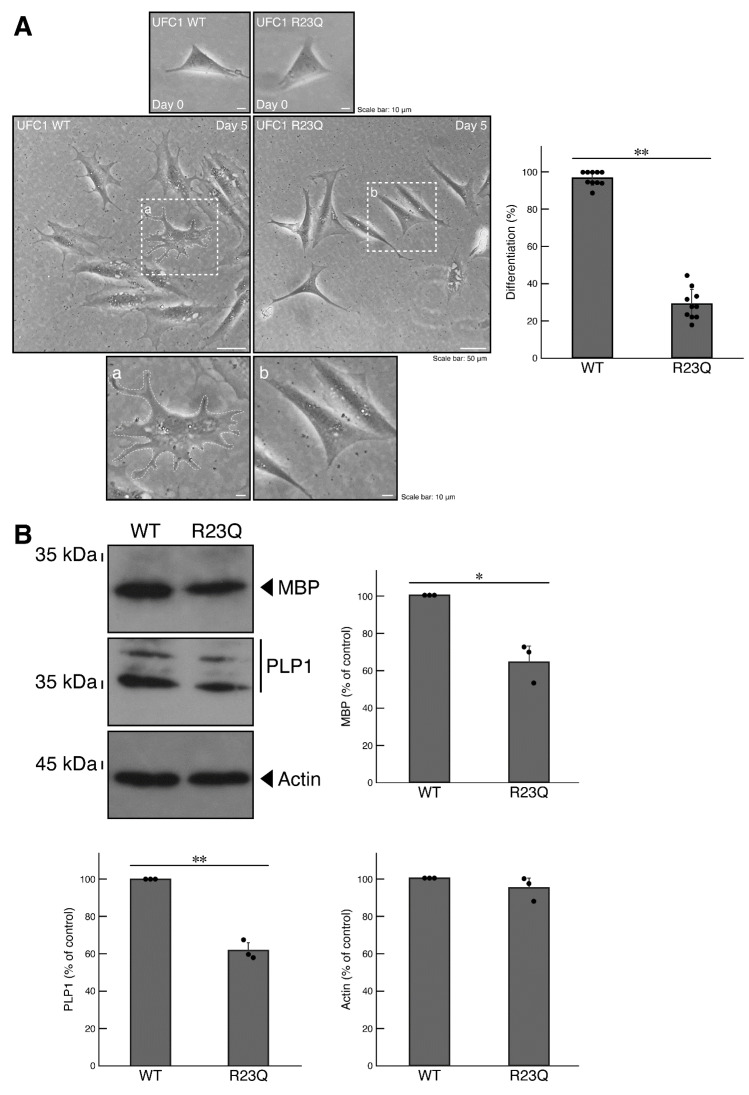
Cells harboring UFC1 with the R23Q mutation fail to undergo morphological differentiation. (**A**) Cells harboring GFP-tagged wild-type (WT) UFC1 or UFC1 (R23Q) were allowed to differentiate for 0 or 5 days. The square fields a and b indicated by the dotted lines in the center panels are magnified in the bottom panels a and b. Cells with widespread membranes were statistically assessed (** *p* < 0.01; *n* = 10 fields [a total of 360 cells per experiment]). (**B**) The lysates of the respective cells were immunoblotted with an antibody against MBP, PLP1, or control actin. Their expression levels were statistically compared to their respective controls (** *p* < 0.01 or * *p* < 0.05; *n* = 3 blots).

We explored the possibility that UFC1 with the R23Q mutation affects differentiation, potentially leading to incomplete differentiation and, consequently, hypomyelination [26]. Using cells harboring UFC1 with the R23Q mutation, we allowed them to differentiate for 5 days. Cells with the R23Q mutation exhibited decreased differentiation, whereas cells with wild-type UFC1 exhibited well-differentiated phenotypes with expanded plasma membranes (Figure 5A). These results were consistent with immunoblotting data using antibodies specific for differentiation/myelination marker proteins (Figure 5B). Cells harboring wild-type UFC1 expressed abundant marker proteins, such as myelin basic protein (MBP) and proteolipid protein 1 (PLP1). In contrast, the internal control actin protein was comparable in both wild-type UFC1 and R23Q-mutation UFC1 cells.

**Figure 6:**
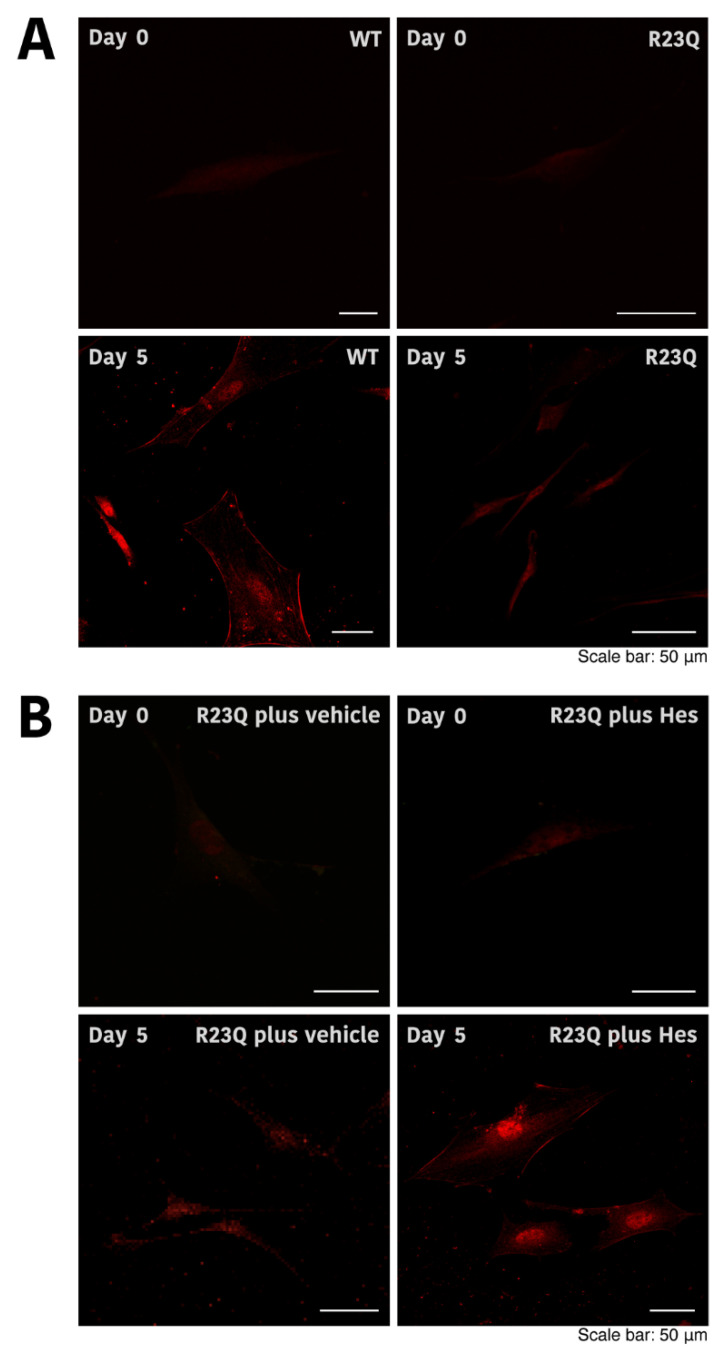
Morphological changes in cells harboring UFC1 with the R23Q mutation and the effect of hesperetin. (**A**,**B**) Cells harboring GFP-tagged wild-type (WT) UFC1 or UFC1 (R23Q) were allowed to differentiate for 0 or 5 days in the presence or absence (vehicle) of hesperetin (Hes). Cells were stained with an anti-PLP1 antibody (red). Representative images are shown.

When cells carrying wild-type UFC1 were stained with an anti-PLP1 antibody, PLP1 antigen levels increased following the induction of differentiation. In contrast, cells harboring UFC1 with the R23Q mutation did not show a significant increase (Panel A, Figure 6).

**Figure 7:**
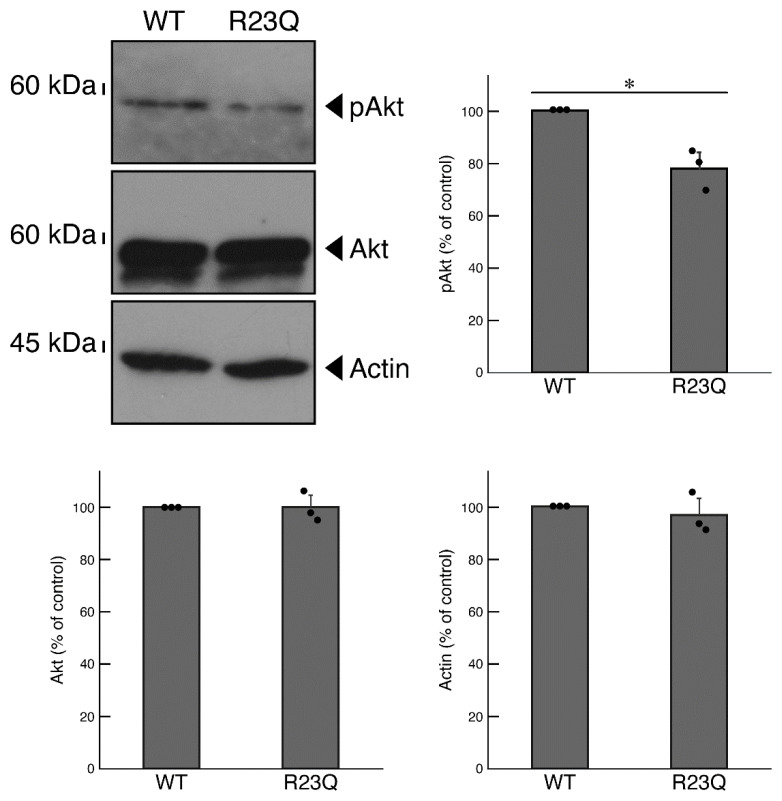
Cells harboring UFC1 with the R23Q mutation show decreased Akt phosphorylation levels. The lysates of cells harboring GFP-tagged wild-type (WT) UFC1 or UFC1 (R23Q) were immunoblotted with an antibody specific for (pS473)Akt (pAkt), Akt, or control actin. Their levels were statistically compared to their respective controls (* *p* < 0.05; *n* = 3 blots).

It is known that Akt is one of the major lysosome signal-associated serine/threonine kinases that controls oligodendroglial cell differentiation and myelination. Abnormal regulation of Akt kinase is linked to defective differentiation and myelination [5,6]. Therefore, we investigated whether the phosphorylation levels of Akt were decreased in cells harboring UFC1 with the R23Q mutation. The results showed decreased Akt phosphorylation levels [5,6], which corresponded to the observed activities in cells harboring UFC1 with the R23Q mutation compared to the wild-type (Figure 7).

### 3.3. Hesperetin Restored Phenotypes Induced by UFC1 with the R23Q Mutation

**Figure 8:**
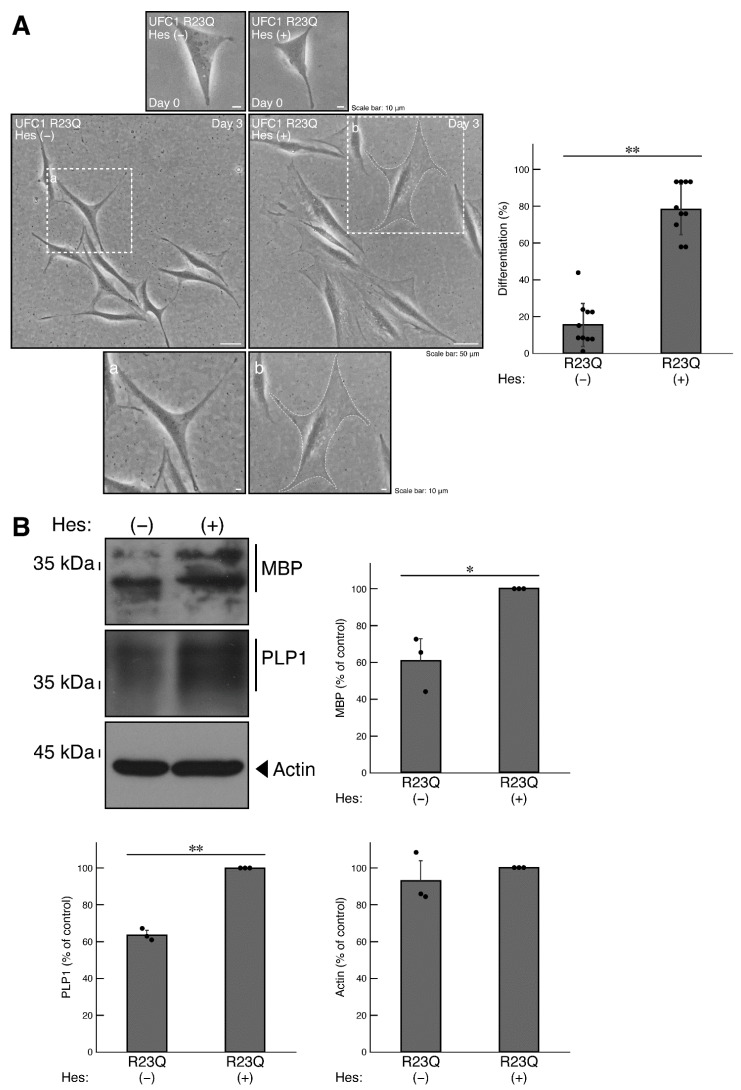
Hesperetin recovers phenotypes in cells harboring UFC1 with the R23Q mutation. (**A**) Cells harboring GFP-tagged UFC1 (R23Q) were allowed to differentiate in the presence (+) or absence (−, vehicle) of 5 micromolar of hesperetin (Hes). The square fields a and b indicated by the dotted lines in the center panels are magnified in the bottom panels a and b. Cells with widespread membranes were statistically assessed (** *p* < 0.01; *n* = 10 fields [a total of 340 cells per each experiment]). (**B**) The lysates of the respective cells were immunoblotted with an antibody against MBP, PLP1, or control actin. Their expression levels were statistically compared to their respective controls (** *p* < 0.01 or * *p* < 0.05; *n* = 3 blots).

**Figure 9:**
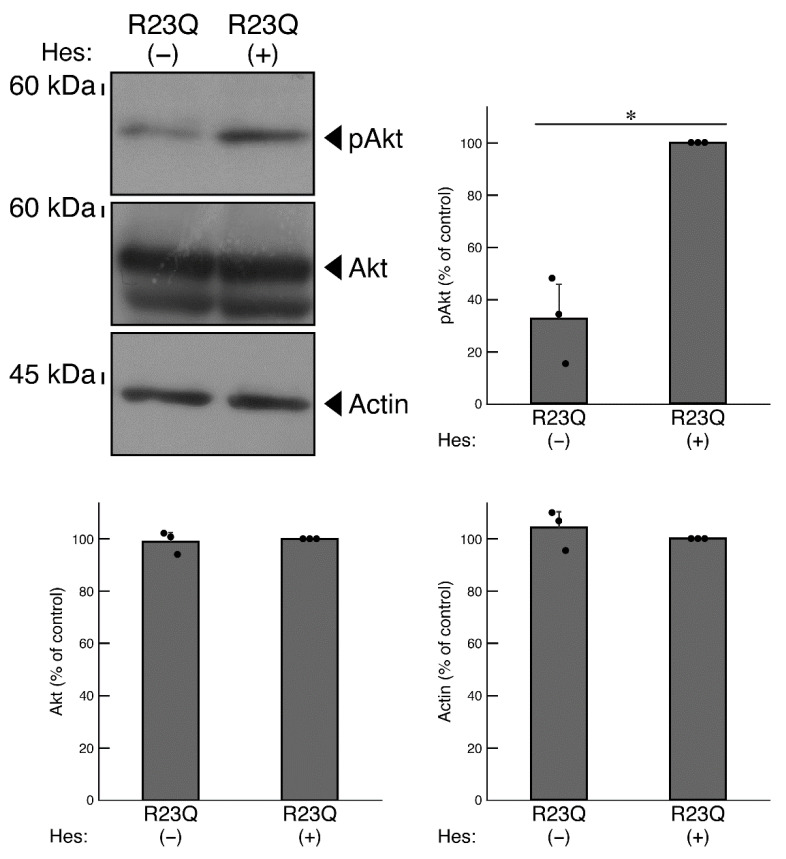
Hesperetin recovers the phosphorylation levels of Akt in cells harboring UFC1 with the R23Q mutation. The lysates of cells harboring GFP-tagged UFC1 (R23Q) in the presence (+) or absence (−, vehicle) of hesperetin (Hes) were immunoblotted with an antibody specific for (pS473)Akt (pAkt), Akt, or control actin. Their levels were statistically compared to their respective controls (* *p* < 0.05; *n* = 3 blots).

Hesperetin, a citrus flavonoid known for its neuroprotective effects in neurological diseases [31,32,33,34,35,36,37,38], was tested for its potential positive effects in cells harboring mutated UFC1. The results revealed that treatment with hesperetin restored the decreased differentiated phenotypes in cells harboring mutated UFC1 (Figure 8A). Along with the phenotypic changes, the expression levels of marker proteins MBP and PLP1 increased (Figure 8B). Similar effects on PLP1 antigen levels were observed in immunostaining (Panel B, Figure 6). In addition, treatment with hesperetin restored the phosphorylation levels of Akt in cells harboring mutated UFC1 (Figure 9).

**Figure 10:**
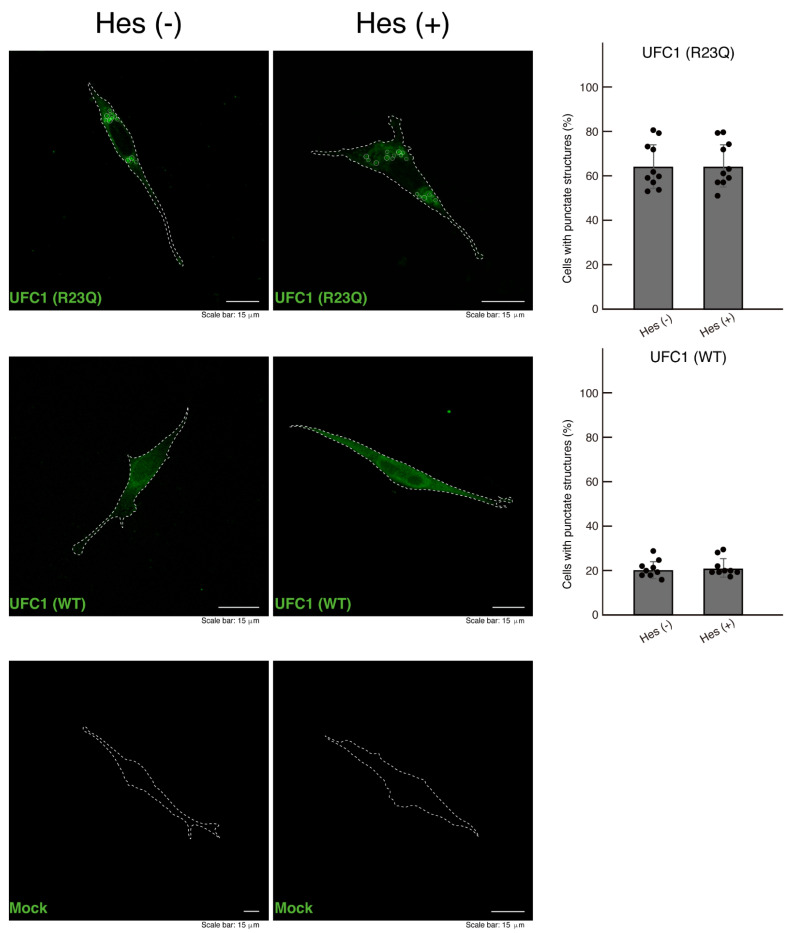
The effect of hesperetin on aggregate-like structures in cells harboring UFC1 with the R23Q mutation. Cells harboring GFP-tagged UFC1 (R23Q) or wild-type (WT, green) or mock-transfected cells were treated with (+) or without (−, vehicle) hesperetin (Hes). Cells with punctate structures (indicated representatively by white circles) were statistically counted (a total of 120 cells per experiment).

We finally investigated whether treatment with hesperetin affects the aggregate-like structures in cells harboring UFC1 with the R23Q mutation. The results showed that hesperetin did not decrease the number of aggregate-like structures in these cells. As a control, no significant changes were observed in the localization of wild-type UFC1 (Figure 10).

Taken together with the cellular and biochemical data presented above, hesperetin may have some protective effects on cells harboring HLD14-related mutant proteins, but the effect appears to be limited.

## 4. Discussion

Post-translational modifications by ubiquitin and ubiquitin-like proteins are crucial for modulating protein functions. UFM1, a member of the ubiquitin-like protein family, covalently binds to target proteins through an array of enzymatic proteins consisting of E1 (activating), E2 (covalently binding), and E3 (ligating) proteins [23]. At the molecular and cellular levels, UFM1 modification, known as UFMylation, plays a key role in mediating protein functions [23]. Dysregulation of the UFMylation system, as observed in studies knocking out or overexpressing molecules involved in UFMylation, disrupts protein homeostasis and causes various intracellular signaling disturbances [23]. These changes are associated with organ and tissue damage, developmental disorders, and several inherited neurological diseases [23,24,25,26,27,28]. For example, the CNS-specific conditional knockout of UFM1 in mice results in neonatal death, accompanied by microcephaly and apoptosis in specific neurons, illustrating that the UFM1 system is essential for CNS development and function [39]. Thus, the complete or partial loss of the UFM1 system, which likely mimics HLD14-like pathologies such as hypomyelination with microcephaly in mice, includes embryologically strong phenotypes [26]. To date, no therapeutic treatment has been identified, even at the mouse model level.

In this study, we report for the first time that UFC1 with the R23Q mutation decreases oligodendroglial cell morphological differentiation and is accompanied by decreased expression levels of differentiation/myelination marker proteins. These cellular phenotypes could be related to the hypomyelinating phenotypes seen in diseases such as HLD14. Conversely, treatment with flavonoid hesperetin can restore these decreased morphological differentiating phenotypes. The phenotypes associated with UFC1 with the R23Q mutation appear to be reversible, at least at the molecular and cellular levels.

We observe that aggregated mutated UFC1 tends to localize in the lysosome, suggesting that the lysosome-resident mutated UFC1 remains undecomposed. This finding is reminiscent of the situation with HLD7- and HLD8-associated mutated proteins of RNA polymerase III subunits A and B (POLR3A and POLR3B), respectively, which are primarily present in the lysosome [12]. In contrast, wild-type POLR3A and POLR3B proteins are indispensable enzymes widely distributed in nuclear and cytoplasmic regions. Similarly, wild-type RNA polymerase I and III subunit C (POLR1C) is localized in the nuclear and cytoplasmic regions, whereas the HLD11-associated mutated protein of POLR1C is primarily localized in the lysosome [12]. It is believed that these mutated RNA polymerase subunits remain undecomposed in the lysosome. This possibility aligns with the observation that Akt signaling through mTOR around the lysosome is markedly down-regulated [5,6,12]. It is of note that decreased Akt phosphorylation is also observed in cells expressing mutated UFC1. From this perspective, the HLD14-related UFC1 mutation appears to be a toxic gain of function.

A potential target for the flavonoid hesperetin could be a molecule within the mTOR signaling pathway. The naturally prenylated flavonoid fraction from *G. glabra* has been shown to stimulate the enzymatic activities of Akt and mitogen-activated protein kinase (MAPK) [36]. Although it is unlikely that hesperetin is present in the *G. glabra* flavonoid fraction, hesperetin or its unidentified metabolic derivatives may stimulate or modulate the enzymatic activities of Akt and kinases related to mTOR in oligodendroglial cells.

Flavonoids, including hesperidin and hesperetin aglycone, do not directly affect the cells and tissues involved in neurodegenerative diseases such as Alzheimer’s disease, Parkinson’s disease, and amyotrophic lateral sclerosis. Instead, they help reduce the neuroinflammation associated with the progression of these diseases [31,32,33]. Despite the unknown critical target of hesperidin and hesperetin, they are also known to slow the progression of these neuropathies [31,32,33]. Hesperetin appears to modulate the activities of certain signaling molecules [34], binding to tyrosine phosphatase 1B (PTP1B) with broad substrate specificity [34]. PTP1B is known to negatively regulate signaling molecules involved with tyrosine-phosphorylated insulin receptor substrate 1 (IRS1) and downstream Akt kinases [36]. Akt-mediated signaling plays an important role in oligodendroglial cell differentiation in vitro, and once differentiation occurs, the signaling cascade continues to act as a central player in the transition to the myelination stage in vivo [5,6]. It is conceivable that modulation of PTP1B activity by hesperetin, acting through Akt signaling, could promote differentiation and myelination. Consequently, hesperetin may help restore the cell conditions observed in neurodegenerative diseases. Although potential hesperetin target molecules like PTP1B are not direct therapeutic targets for underlying diseases such as HLD14, hesperetin may promote oligodendroglial cell differentiation in pathological conditions, helping to improve their condition at least at the molecular and cellular levels.

In an artificial demyelination model in the rodent optic chiasm, which serves as the CNS, treatment with hesperetin has been shown to decrease damage to the myelin sheath [37,38]. This beneficial effect is attributed to hesperetin’s regulation of glial morphogenesis. It is likely that hesperetin has the ability to ameliorate certain risk factor molecules involved in the pathological conditions that drive demyelination and/or hypomyelination.

In a *Drosophila* model, the disruption of genes in the UFM1 cascade, including UFC1, greatly decreases the number of neuroblasts, resulting in the smaller brain size observed in microcephaly [40]. It appears that molecules within the UFM1 cascade are responsible for changes in the phosphorylation level of Tyr-15 on cyclin-dependent kinase 1 (CDK1), a negative cell cycle regulator of the G2 to M transition [40]. Since the UFM1 cascade regulates cell cycle progression at the entry into mitosis, certain chemicals or drugs targeting CDK1 could be used to address microcephaly phenotypes, as observed in the case of HLD14 [23,24,25,26,27,28]. Simply replacing the damaged genes or their products may improve pathological conditions; however, the situation is not always straightforward. In cells lacking UBA5 or UFC1, re-expression of UBA5 results in the inability of cells to migrate to a suitable position [41]. In contrast, co-expression of UBA5 with UFM1 restores this ability [41]. Since the expression levels of UFMylation enzymes are tightly regulated to ensure proper directionality of UFM1 transfer, compensating for deficiencies in the UFMylation system in cells and tissues may present challenges.

The UFMylation system is known to be involved in the internalization of the 140 kDa neural cell adhesion molecule (NCAM140) from the cell surface [42]. While the specificities for other NCAM isoforms, such as NCAM120 and NCAM180, remain unknown, the UFMylation system interacts with the intracellular domain of transmembrane proteins, like NCAM140 [42]. In the ubiquitin cascade, the interaction of ubiquitin with the intracellular domain of transmembrane proteins often leads to their degradation [43,44,45,46], but it is unlikely that the UFMylation system is involved in the protein degradation of NCAM140. Since differentiating or myelinating oligodendroglial cells biosynthesize various cell adhesion molecules and receptors [1,2,3,4], it is possible that the UFMylation system contributes to the recycling and possible degradation of transmembrane proteins, thereby triggering oligodendroglial cell differentiation and subsequent myelination.

Unlike ubiquitination, UFMylation is not necessarily involved in regulating protein clearance and homeostasis [43,44,45,46]. Although ubiquitination also triggers positive signals to activate the intracellular signaling system, its effect is very limited [43,44,45,46]. It remains to be established whether UFMylation plays a positive or negative role in protein clearance and homeostasis and whether UFMylation acts as a central player or a secondary contributor to the cellular signaling system. However, it is evident that dysregulation of the UFMylation system is associated with defective protein homeostasis and disruptions in intracellular signaling.

In this study, we demonstrate that the HLD14-related UFC1 mutant protein primarily localizes as aggregates in the lysosome, leading to decreased morphological differentiation. Treatment with hesperetin restores these undifferentiated states. Further studies will advance our understanding of not only the detailed mechanism by which the mutated UFC1 causes decreased cell morphogenesis in genetically modified mice and primary cells but also how hesperetin restores these phenotypes. Such studies will enable the development of therapeutic target–specific medicines for diseases related to the UFMylation system, including HLD14.

## Figures and Tables

**Table 1:** Key materials used in this study.

Reagents or Materials	Companies or Sources	Cat. Nos.	Lot. Nos.	Concentrations Used
Key antibodies				
Anti-Lys-Asp-Glu-Leu (KDEL)	MBL (Tokyo, Japan)	M181-3	004	Immunofluorescence (IF), 1:200
Anti-GM130	BD Biosciences (East Rutherford, NJ, USA)	610823	8352796	IF, 1:200
Anti-lysosomal-associated membrane protein 1 (LAMP1)	Santa Cruz Biotechnology (Santa Cruz, CA, USA)	sc-20011	J0919	IF, 1:100
Anti-green fluorescent protein (GFP)	MBL	598	084	Immunoblotting (IB), 1:100,000
Anti-myelin proteolipid protein 1 (PLP1)	Atlas Antibodies (Stockholm, Sweden)	HPA004128	8115828	IB, 1:1000; IF, 1:250
Anti-myelin basic protein (MBP)	BioLegend (San Diego, CA, USA)	836506	B225469	IB, 1:500
Anti-actin (also called pan-bata type actin)	MBL	M177-3	007	IB, 1:5000
Anti-Akt	Cell Signaling Technology (Danvers, MA, USA)	4691T	28	IB, 1:100
Anti-phospho-Akt (pS473 Akt)	Cell Signaling Technology	4060S	27	IB, 1:500
Anti-UFC1	Proteintech (Rosemont, IL, USA)	15783-1-AP	94634	IF, 1:200
Anti-IgG (H+L chain) (rabbit) pAb-HRP	MBL	458	354	IB, 1:5000
Anti-IgG (H+L chain) (mouse) pAb-HRP	MBL	330	366	IB, 1:5000
Alexa Fluor TM 488 goat anti-mouse IgG (H+L)	Abcam (Cambridge, UK)	ab150113	GR158438	IF, 1:500
Alexa Fluor TM 594 goat anti-mouse IgG (H+L)	Thermo Fisher Scientific (Waltham, MA, USA)	A11005	226-8383	IF, 1:500
Alexa Fluor TM 488 goat anti-rabbit IgG (H+L)	Thermo Fisher Scientific	A11008	075-1094	IF, 1:500
Alexa Fluor TM 594 goat anti-rabbit IgG (H+L)	Thermo Fisher Scientific	A11012	201-8240	IF, 1:500
Chemicals				
Hesperetin	Santa Cruz Biotechnology	sc-202647	D1921	Final concentration, 5 microM
Dimethyl sulfoxide (DMSO)	FUJIFILM Wako Pure Chemical Corporation (Tokyo, Japan)	047-29353	CDN0170	Final concentration, less than 0.1%
Recombinant DNAs				
pEGFP-C1-UFC1	PCR-based amplificated human UFC1 was ligated into pEGFP-C1 in this study	n.d.	n.d.	1.25 microgram of DNA per 3.5 cm dish or 6 cm dish
pEGFP-C1-UFC1 (R23Q)	The pEGFP-C1-UFC1 was mutated using PCR-based technique in this study	n.d.	n.d.	1.25 microgram of DNA per 3.5 cm dish or 6 cm dish
pEGFP-C1-UFC1 (T106I)	The pEGFP-C1-UFC1 was mutated using PCR-based technique in this study	n.d.	n.d.	1.25 microgram of DNA per 3.5 cm dish or 6 cm dish
pEGFP-C1 (for mock transfection)	Addgene (Watertown, MA, USA)	Vector database No. 2487	n.d.	1.25 microgram of DNA per 3.5 cm dish or 6 cm dish

## Data Availability

The datasets used and/or analyzed for the current study are available from the corresponding author upon reasonable request.

## Supplementary Materials

The following supporting information can be downloaded at https://www.mdpi.com/article/10.3390/medicines12010002/s1, Figure S1. The wild-type UFC1 protein is present throughout the cytoplasm. (A,B) FBD-102b cells were transfected with the plasmid encoding GFP-tagged wild-type UFC1. Cells were detected with transfected proteins (green) and the organelle antigen (red). Scan plots were performed along the white dotted lines in the direction of the arrows in the color images (green and red ones); Figure S2. The R23Q or T106I proteins exhibit polymeric molecular weight structures in non-denaturing polyacrylamide gel electrophoresis. (A,B) The lysates of cells transfected with the plasmid encoding GFP-tagged wild-type (WT) UFC1, UFC1 (R23Q), or UFC1 (T106I) were subjected to non-denaturing (A, upper images) and denaturing (D, lower images) polyacrylamide gel electrophoresis and detected by immunoblotting specific for GFP. The position corresponding to the molecular weight of the wild-type monomer or the R23Q or T106I monomeric or polymeric structures (including dimer and high molecular weight products) is shown in the large-size gels. Immunoreactive bands between monomers and dimers are predicted to be post-translationally modified; Figure S3. Original size gels saved as TIFF files for Figure 5. Each lane is labeled; Figure S4. Original size gels saved as TIFF files for Figure 7. Each lane is labeled; Figure S5. Original size gels saved as TIFF files for Figure 8. Each lane is labeled; Figure S6. Original size gels saved as TIFF files for Figure 9. Each lane is labeled.
