# Supplementary material for: Hypomyelinating Leukodystrophy 14 (HLD14)-Related UFC1 p.Arg23Gln Decreases Cell Morphogenesis: A Phenotype Reversable with Hesperetin
Source: Medicines (Basel). 2025 Jan 16;12(1):2. doi: 10.3390/medicines12010002 (PMC11755592; doi:10.3390/medicines12010002)
Supplement: Supplementary file 1 [file medicines-12-00002-s001.zip › Supplemental figure legends.pdf]

**Supplemental figure legends**

Figure S1. The wild type UFC1 protein is present throughout the cytoplasm. (A, B) FBD-102b cells were transfected with the plasmid encoding GFP-tagged wild type UFC1. Cells were detected with transfected proteins (green) and the organelle antigen (red). Scan plots were performed along the white dotted lines in the direction of the arrows in the color images (green and red ones).

Figure S2. The R23Q or T106I proteins exhibit polymeric molecular weight structures in non-denaturing polyacrylamide gel electrophoresis. (A, B) The lysates of cells transfected with the plasmid encoding GFP-tagged wild type (WT) UFC1, UFC1 (R23Q), or UFC1 (T106I) were subjected to non-denaturing (A, upper images) and denaturing (D, lower images) polyacrylamide gel electrophoresis and detected by immunoblotting specific for GFP. The position corresponding to the molecular weight of the wild type monomer or the R23Q or T106I monomeric or polymeric structures (including dimer and high molecular weight products) is shown in the large-size gels. Immunoreactive bands between monomers and dimers are predicted to be post-translationally modified.

Figure S3. Original size gels saved as TIFF files for Figure 5. Each lane is labeled.

Figure S4. Original size gels saved as TIFF files for Figure 7. Each lane is labeled.

Figure S5. Original size gels saved as TIFF files for Figure 8. Each lane is labeled.

Figure S6. Original size gels saved as TIFF files for Figure 9. Each lane is labeled.
