# Supplementary figures and images for: Hypomyelinating Leukodystrophy 14 (HLD14)-Related UFC1 p.Arg23Gln Decreases Cell Morphogenesis: A Phenotype Reversable with Hesperetin
Source: Medicines (Basel). 2025 Jan 16;12(1):2. doi: 10.3390/medicines12010002 (PMC11755592; doi:10.3390/medicines12010002)

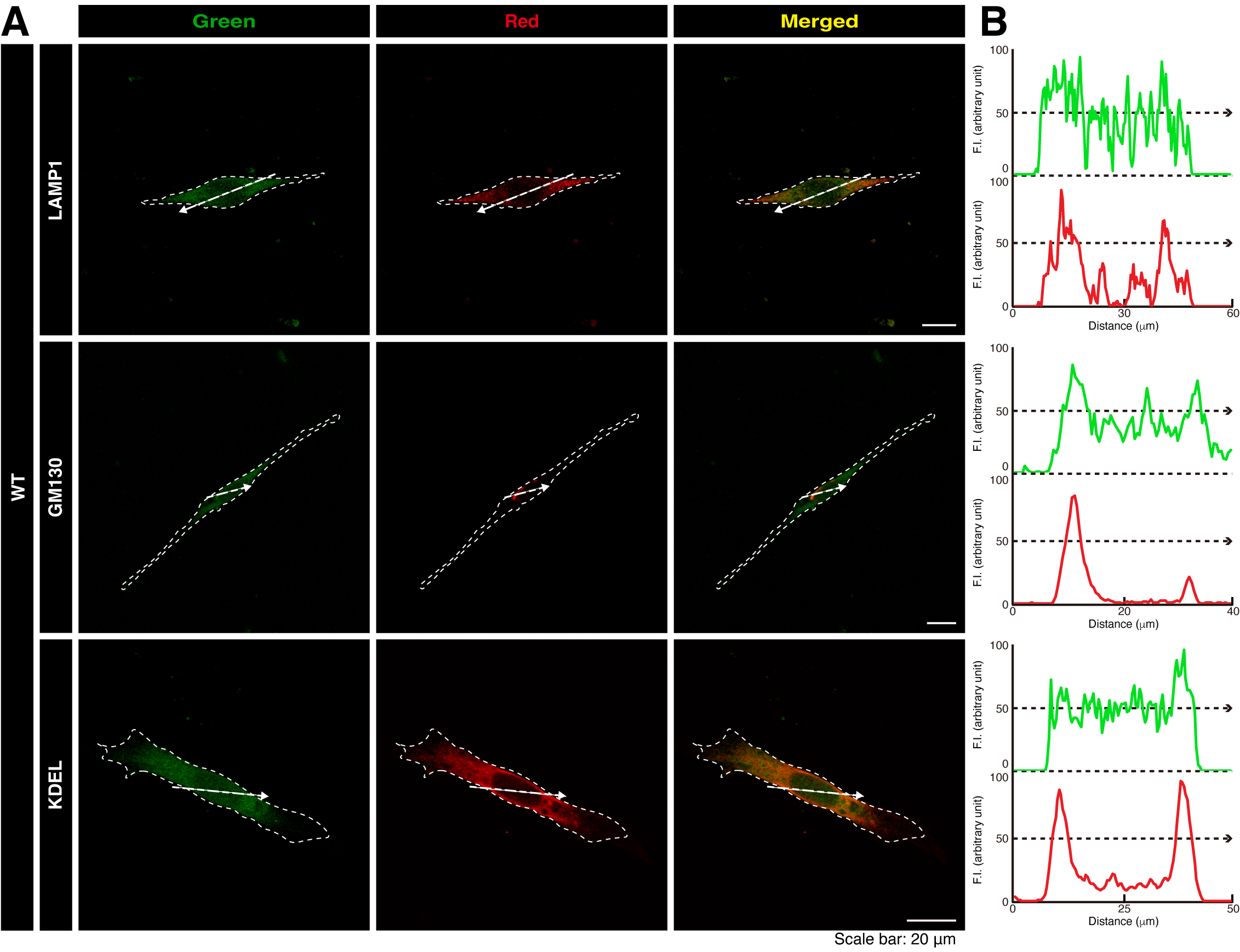

Supplement: Supplementary file 1 [file medicines-12-00002-s001.zip › Figure S1.tif]

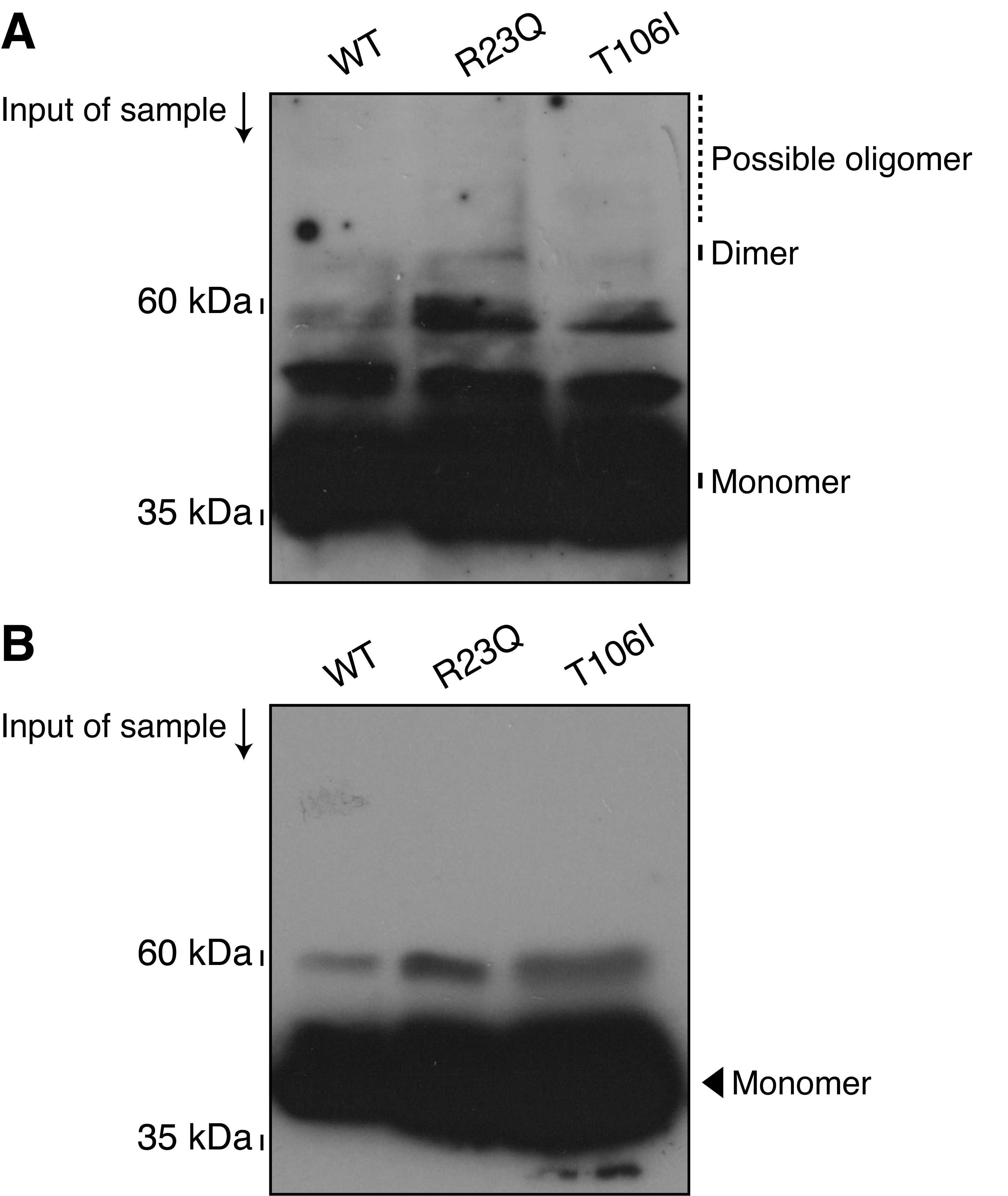

Supplement: Supplementary file 1 [file medicines-12-00002-s001.zip › Figure S2.tif]

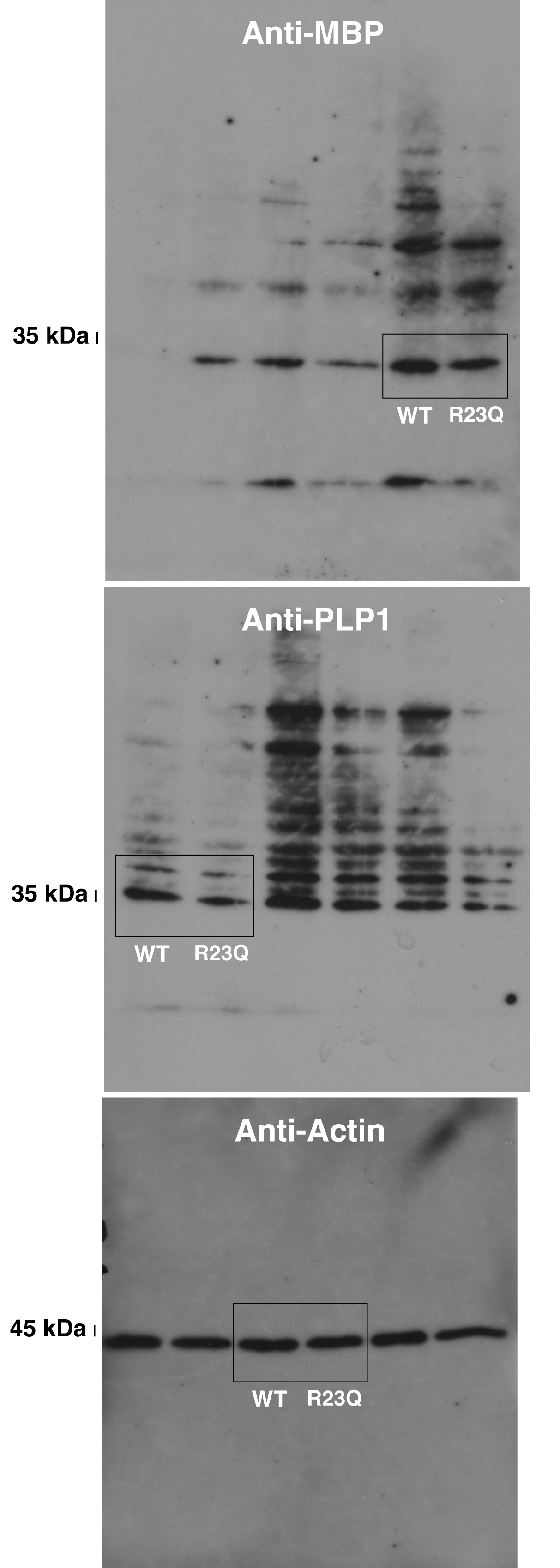

Supplement: Supplementary file 1 [file medicines-12-00002-s001.zip › Figure S3.tif]

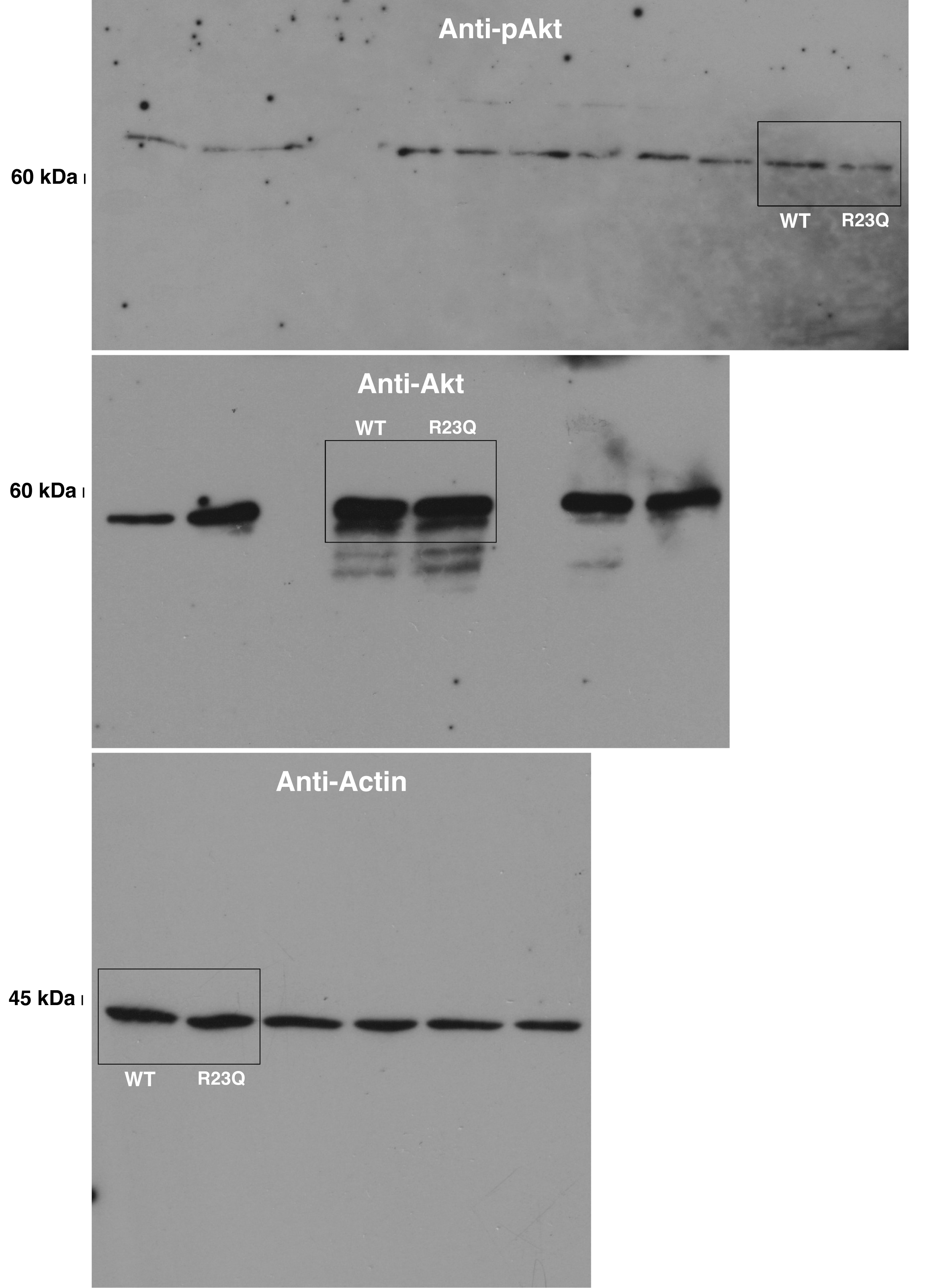

Supplement: Supplementary file 1 [file medicines-12-00002-s001.zip › Figure S4.tif]

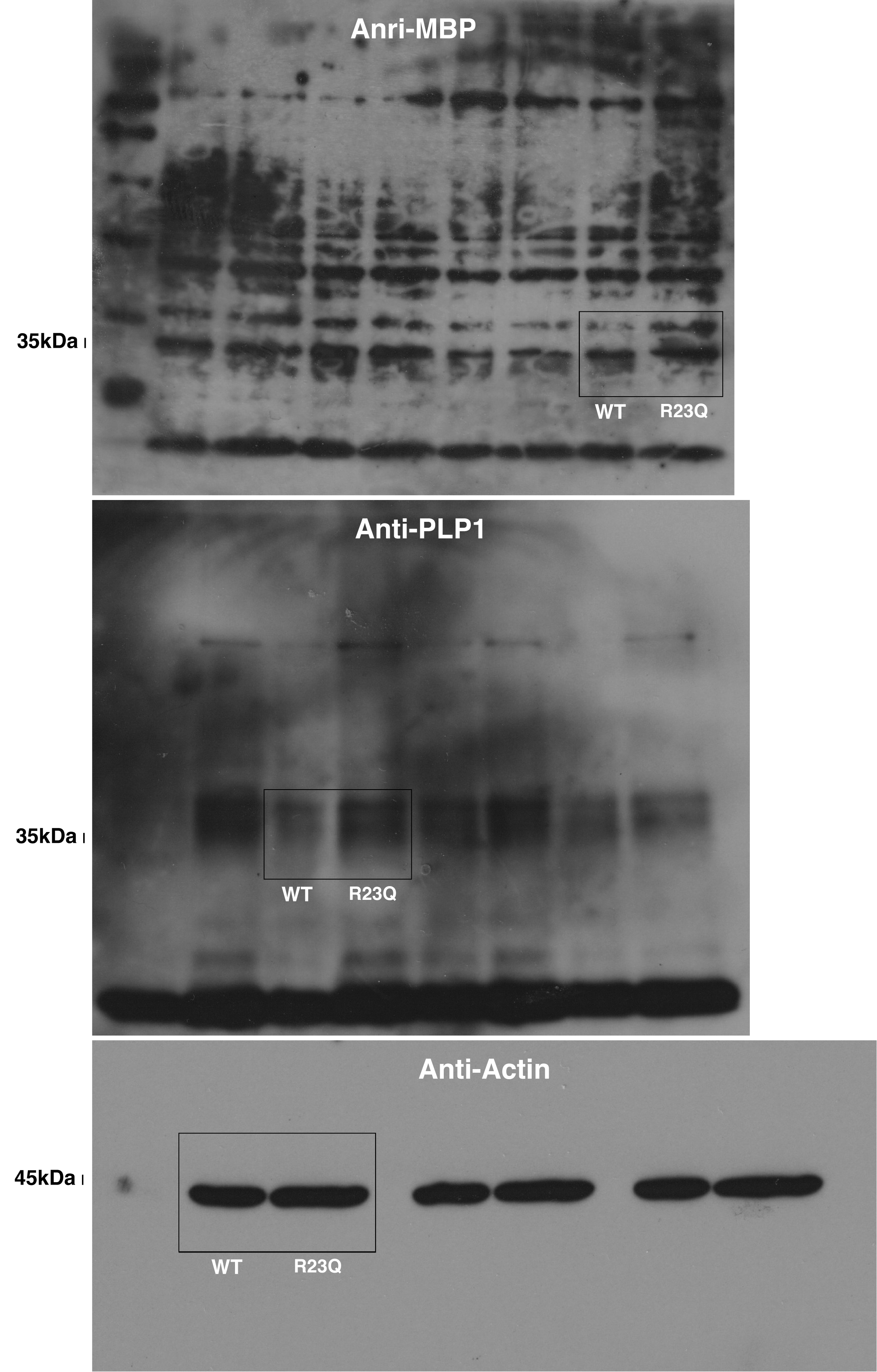

Supplement: Supplementary file 1 [file medicines-12-00002-s001.zip › Figure S5.tif]

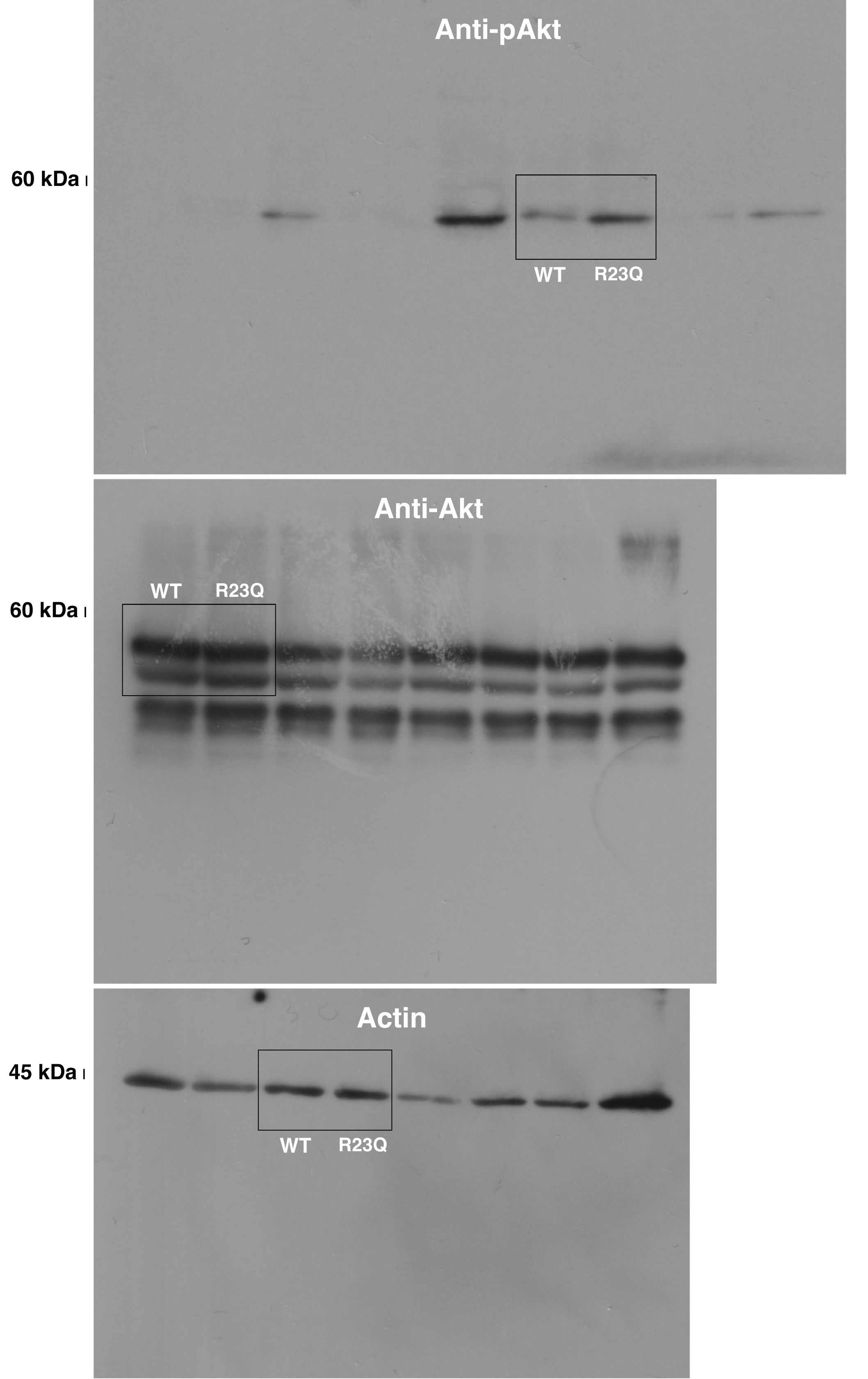

Supplement: Supplementary file 1 [file medicines-12-00002-s001.zip › Figure S6.tif]
